# Understanding age and society using natural populations

**DOI:** 10.1098/rstb.2022.0469

**Published:** 2024-10-28

**Authors:** Josh A. Firth, Gregory F. Albery, Sandra Bouwhuis, Lauren J. N. Brent, Roberto Salguero-Gómez

**Affiliations:** ^1^ Faculty of Biological Sciences, University of Leeds, Leeds, UK; ^2^ Department of Biology, Oxford University, Oxford, UK; ^3^ School of Natural Sciences, Trinity College Dublin, Dublin, Republic of Ireland; ^4^ Department of Biology, Georgetown University, Washington, DC, USA; ^5^ Institute of Avian Research, Wilhelmshaven, Germany; ^6^ Centre for Research in Animal Behaviour, University of Exeter, Exeter, UK

**Keywords:** sociality, ageing, social networks, behavioural ecology, contagions, senescence

## Abstract

Ageing affects almost all aspects of life and therefore is an important process across societies, human and non-human animal alike. This article introduces new research exploring the complex interplay between individual-level ageing and demography, and the consequences this interplay holds for the structure and functioning of societies across various natural populations. We discuss how this Special Issue provides a foundation for integrating perspectives from evolutionary biology, behavioural ecology and demography to provide new insights into how ageing shapes individuals’ social behaviour and social associations, and how this in turn impacts social networks, social processes (such as disease or information transfer) and fitness. Through examining these topics across taxa, from invertebrates to birds and mammals, we outline how contemporary studies are using natural populations to advance our understanding of the relationship between age and society in innovative ways. We highlight key emerging research themes from this Special Issue, such as how sociality affects lifespan and health, the genetic and ecological underpinnings of social ageing and the adaptive strategies employed by different species. We conclude that this Special Issue underscores the importance of studying social ageing using diverse systems and interdisciplinary approaches for advancing evolutionary and ecological insights into both ageing and sociality more generally.

This article is part of the discussion meeting issue ‘Understanding age and society using natural populations ’.

## 1. Introduction

All social systems are fundamentally governed by the states of the individuals within them and the social connections between these individuals [1]. In this context, individual age provides a particularly relevant state to consider, as age shapes almost all aspects of organisms’ lives across diverse systems [2], including their social behaviour [3,4]. In this way, the interplay between individual age and sociality can be fundamentally expected to hold varied consequences for individuals, groups and the wider population they are embedded within [4,5]. This Special Issue is aimed at increasing our understanding of ‘age and society using natural populations’ by integrating the study of ecology, evolutionary biology, behaviour and demography across a broad range of study systems and using diverse approaches. Along with developing our understanding of social systems, this integrative approach aligns with recent calls across disciplines for a systems approach to ageing biology, highlighting the importance of considering multiple interacting factors when considering ageing as a complex process influenced by dynamic interactions across various biological levels and environmental contexts [6,7].

In this introduction, we introduce the novel interdisciplinary research on age and sociality across animal systems held in this Special Issue and outline important advances as well as key challenges. Specifically, we begin with justifying the use of animal systems for examining sociality and age (§2), before moving to discussing the empirical and theoretical advances in understanding sociality and ageing (§3) by highlighting the potential mechanisms underpinning social ageing and differential contributions of the genetic, environmental and social factors driving these changes (§3a), exploring how these individual-level age-related factors scale up to influence societal structure and ecological outcomes (§3b), and their consequences for fitness and evolutionary outcomes (§3c). We also then identify current challenges and considerations in dissecting the age-sociality interplay, and propose key areas for future theoretical and applied research (§4). Finally, in §5, we recap how the Special Issue provides a new comprehensive foundation for understanding how age and sociality interact across species and contexts, offering new directions for both theoretical and applied research.

### 2. Why animal systems for examining sociality in relation to age?

A key link between age and society is captured in the concept of social ageing [4,5,8], which can broadly be defined as changes in individuals’ social connections that occur as they age. Previous reviews have outlined the operation of social ageing in relation to drivers of late-life changes in social behaviour across mammals [4]. In fact, social ageing can be considered for all social species, as it encompasses various dimensions including alterations in social behaviour [9], social roles [10], types of interactions [11] and opportunities to interact [12–14], as well as the overall structure of social networks individuals are embedded within [5], and across all life stages from early development to later-life. Broadly, social ageing can be considered not only at the level of the individual [4], but also at the level of the group or the population as a whole [15–18], and gaining a fundamental understanding therefore requires knowledge of processes across these scales. Populations across the animal kingdom offer various opportunities to contribute to this growing body of knowledge, particularly due to the availability of (i) long-term studies [19–21] and potential for (ii) experiments [22–24] and (iii) cross-system insights and comparisons [2,4,25].

First, many long-term studies simultaneously track individuals’ states and social associations throughout their lives [26], and even across generations [19]. As such, these populations can often be considered as model systems for not only just studying ageing in natural environments [2], but also for social ageing specifically [4,5]. Indeed, understanding ageing within societies is somewhat inextricably linked to long-term studies, almost by definition, as studies have to track the same individuals over their life courses—and also as ‘ageing’ is not a treatment that can be applied as such, and rather must manifest naturally over time. As such, long-term studies provide unique and invaluable data that can be used to obtain ecologically valid insights on how age affects social behaviour and structure over time, as called upon in this Special Issue [8,27,28]. For example, this issue shows how long-term research on rhesus macaques (*Macaca mulatta*) has revealed intriguing links between early life conditions, longevity, social associations and disease [8,29]. Similarly, long-term studies on wild deer (*Cervus elaphus*) are highlighting how age-related changes in social networks can influence exposure to disease [28]. As well as adding social ageing perspectives into established model systems, emerging model systems can extend and expand perspectives on social ageing. For example, white-faced capuchins add to the so-far limited number of ageing models that exhibit multi-faceted social structures and long lifespans [30]; ants experience social ageing at the colony level and offer unique insights into how ageing affects social roles and how this scales up to the broader system [15]; and common terns (*Sterna hirundo*) and house-sparrows (*Passer domesticus*) now provide new systems in which pedigree data can be combined with social data to detect changes in sociality across the life course on both the phenotypic and genetic levels [8,27]. Indeed, across different taxa, and even including species less traditionally thought of as ‘social’ [30,31], longitudinal studies are shedding light on the diverse ways ageing impacts social connections across individuals’ lifetimes and across generations. These systems and perspectives will be increasingly useful for understanding how social ageing operates across the spectrums of social organization (from primarily non-social relatively solitary species to highly social communal living species) and life histories (e.g. from short-lived, fast- paced to long-lived, slow-paced) [5,32].

Second, animal systems offer insights into social ageing through the unique potential to carry out experiments. In this Special Issue, Harrison *et al*. [31] use a systematic literature review to demonstrate how laboratory studies on insects can reveal how social environments, including manipulatable factors like group size and opposite-sex exposure, can significantly influence lifespan and ageing, and thus highlight how these can be used to address important gaps in our understanding of ageing. Moreover, novel experimental manipulations, such as altering the age composition of populations, can causally demonstrate how age structure shapes social behaviour and fitness. For example, Cook *et al*. [33] used an experimental approach in forked fungus beetles to test how population age structure influences social network structure and its consequences for reproductive success. While social ageing experiments with captive invertebrates currently stand out as an accessible approach, there is also the possibility to extend experimental approaches into the wild. Experimental manipulations within long-term study systems of wild birds, for instance, appear particularly feasible and could yield valuable insights [5,34]. Finally, due to the long-term study of particular populations, various opportunities for ‘natural experiments’ can arise, and future work may be able to assess phenomena such as how age may influence how macaque social systems respond to ecological disturbances [8,29], how outbreaks of highly pathogenic avian influenza may depend on, and shape, the social interactions of common terns depending on their age [27], or even how the COVID-19 pandemic shaped social systems of long-term populations due to changes in anthropogenic activities [35,36].

Third, animal populations offer the ability to go beyond insights into individual systems of set social and age structures and allow for cross-system analyses which will be notably important for establishing the broader patterns that govern social ageing and the interplay between age and society across contexts [4]. Indeed, while systematic literature analyses offer a first step towards developing a theoretical grounding of social ageing across species [5,31], new comparative approaches are becoming increasingly feasible and powerful due to the amount of life history data available for many species. In this issue, such data are used for 152 species (from jellyfish to humans) along a continuum of sociality—from solitary to tightly social— to test how sociality correlates with key demographic properties [32]. As the number of systems with fine-scale social network data increases [5,37], meta-datasets of social network data (e.g. [21,37,38])—combined with demographic data across species—will provide an ideal framework for understanding how sociality, ageing and demography vary across environmental conditions. Aside from these comparative approaches, general mathematical modelling of sociality and age currently offers a particularly needed approach for grasping the expectations surrounding the fundamental mechanisms underlying social ageing. For example, in this Special Issue, Mann [39] explores how rational agents are expected to prioritize long-term rewards over their life course, demonstrating that individuals may make less rewarding decisions in the short term to maintain group cohesion for later in life, providing insight into collective decision making in ageing societies. Mathematical models can also enable insights into the potential consequences surrounding ageing in social systems. For instance, also in this Special Issue, Hasenjager & Fefferman [40] show how older individuals, through the formation of tight-knit social groups within their networks, may enhance their capacity to transmit information, which will be exaggerated even further in scenarios resembling complex contagions [40,41]. Generative models are underexploited tools in social network ecology [42–44], and they could offer an interesting solution to the problem of the need for long-term studies in social ageing specifically [45].

## 3. Empirical advances in understanding sociality and ageing

### (a) Mechanisms

Empirical studies are increasingly revealing age-related patterns of social behaviour across diverse taxa. This Special Issue includes empirical research on primates [8,29,46], ungulates [28], insects [30,31] and birds [8,27,34] that demonstrates that age may influence social interactions in diverse ways. Together, these studies build on the more extensive body of work on age-specific traits in wild populations, such as ornamentation, phenology, morphology, physiology, reproductive performance and survival [2,47–55]. Once such patterns are observed, a key question emerges: what biological processes drive these changes?

Age-specific phenotypes can result from within-individual changes as organisms age. Alternatively, however, they may stem from selective disappearance, where individuals with certain phenotypes are more likely to be lost from the population over time (e.g. [56,57]). Distinguishing between these two possibilities requires detailed longitudinal data, which makes it possible to disentangle within-individual ageing from selective disappearance [58,59]. Indeed, selective disappearance alone can lead to older individuals being either more or less social than their younger counterparts [4,5]. For instance, the least social individuals may face higher mortality if social connections are important for resource acquisition (e.g. [60]) or protection from predators (e.g. [61]). Conversely, those who are less social might survive longer if sociality increases competition [27] or exposure to infectious diseases [8,28]. In this way, even without within-individual social ageing, there would be an apparent difference in the average sociability of older individuals compared with younger individuals.

When considering within-individual processes of social ageing, a key question is why and how social behaviour changes as individuals age. A summary of these potential mechanisms and predicted patterns of behaviour for individuals beyond their developmental years can be found in Siracusa *et al*. [4]. More recently, the number of studies providing evidence for social ageing mechanisms has grown relatively rapidly, including studies in this Special Issue. For example, one potential mechanism is a relatively passive one, emerging from the fact that the sociability of an individual is not just given by their own propensity for social interactions, but is also a result of the surrounding individuals [62]. In this case, changes in sociality with age may be underpinned by individuals losing social contacts due to mortality (e.g. [8]) or the retaining of long-term mates after an initial mate-selection phase (e.g. [63–65]). Alternatively, older individuals may become more central in social networks due to their accumulated knowledge, acting as repositories of information and potentially being a more attractive social associate [40]. However, they may also be actively excluded from social groups (e.g. [66,67]), possibly because of shifts in their spatial behaviour [14]. Another potential driver of change is adaptive avoidance, where older individuals reduce social interactions to lower the risk of mortality linked to immunosenescence [8,28,68].

On a more physiologically driven mechanistic level, several biological factors may drive changes in social behaviour as animals age [4]. These—for example—include shifts in cognitive ability [69], body size [70,71], immune function [72,73], mitochondrial performance [74] and hormone levels and energetic status [46]. Such factors may influence how individuals interact as they grow older, potentially affecting their social standing or overall behaviour. However, studies that directly link changes in these physiological traits to social behaviour remain scarce, despite being critical for advancing our understanding of the mechanisms underlying social ageing [30]. In addition to these physiological drivers, exploring epigenetic [75,76], molecular [77] and neural [78,79] pathways is another key avenue for research into the ageing phenotype, including its social dimensions. New technologies, such as advanced sequencing techniques [80–82], now allow researchers to delve deeper into these molecular processes. Pedigree data may also offer new opportunities to explore whether indirect genetic effects contribute to social ageing [27].

To complement detailed examinations of social ageing mechanisms within specific model systems, we can also explore how relatively simple behavioural rules might underpin general social ageing phenotypes. For example, social selectivity, whereby ageing individuals maintain fewer social connections and become more selective and focus on key social relationships, has been documented in humans [83] and other animals [8,84,85]. A simple behavioural rule that may underpin the social selectivity phenotype is that individuals may have a propensity to associate with others based on their prior knowledge or interactions; this rule would then mean that individuals begin life by broadly sampling their social environment (as no prior associations exist), leading younger individuals having more unique connections than older ones. Over time, however, these younger individuals are likely to resample their previous connections, and thus increase their partner selectivity as an outcome of repeated social interactions over time. Considering how such rules may generate the relatively complicated patterns we often observe in temporal social networks as individuals age will now be particularly useful [39,44].

Once we have identified the general mechanisms of social ageing, the following challenge is to investigate whether—and if so, why—individuals vary in their rates of social ageing [27,30]. A further challenge is then to identify how these differences affect fitness outcomes [29,30]. Understanding these individual differences will be crucial for revealing the micro-evolutionary processes that shape social traits in natural populations.

### (b) Societal and ecological outcomes of ageing

Beyond the consequences of social ageing for the individual, there is a wide range of emergent consequences at the group and population level [5]. Most notably, because social network structure is the product of individual-level processes [1], age-related within-individual changes in behaviour are expected to shape emergent higher-level social structures [15,34]. Additionally, because social structure is tightly linked to individual health and success [67,86], these processes can feed back to shape outcomes for those involved [5,8,40]. Connecting these two layers of processes in a bidirectional framework is vital for addressing a long-standing gap in understanding the evolutionary mechanisms governing the ecological consequences of social ageing.

Competition and cooperation are two social processes that are often central to many societies’ ecology [81,87], and their balance is likely to change substantially with age. Various contributions in this Special Issue point to how social ageing can influence the strength and direction of competition and cooperation between individuals by altering their interactions [5,30,32,46]. For example, older individuals might lack the energy to actively invest in others to the same extent and so may take more and reciprocate less [46], so social networks in aged populations may be skewed away from cooperation which will influence their functioning. Currently, there is also much interest in how social behaviours that vary with age can influence contagions in networks [8,28,40]. For example, if ageing reduces social connections, then social networks composed of a greater number of older animals might be less well-connected than younger networks [5,15], curbing the flow of socially transmitted pathogens [8]. Although the same may be expected for the spread of socially transmitted information, the actual transmission of socially learnt behaviours may depend on more than just social connections alone [40,41,88]. As such, how we expect behaviours to spread in this context, especially if the expression or learning of the behaviour is age-dependent, remains largely unknown. A future research priority should be to understand the phenotypes and mechanisms involved in social contagions [41,88,89], alongside the age-structured networks that emerge through a variety of mechanisms, to inform whether these networks should promote or reduce spread [40], and how this changes over time [39].

Through determining the balance of costs of sociality (infection [8,28], competition [34], etc.) versus the benefits (cooperation [87], learning [40], group foraging [60]), ecological processes embedded with animal societies may be expected to influence fitness [81]. Because of the correlated and multidimensional nature of these processes, it is now important to conduct experiments to disentangle them, yet such experiments in natural populations have previously been difficult [23,24]. Nevertheless, this Special Issue contains various proposals outlining how animal systems, particularly insect systems, may be particularly useful for experiments which directly test the outcomes of specific social processes [15,30,31].

One advantage of increasing understanding of how individual-level social ageing scales up to shape population-level social processes is that this knowledge may help in translating fundamental insights into applied practices. For example, expanding the reach and generality of current models may allow us to apply our knowledge of sociality and ageing to improve conservation efforts, possibly allowing the development of strategies for mitigating age-related declines in endangered or threatened species [5]. This may be particularly important in ageing populations, e.g. if birth rates are declining and resulting in skewed demographic pyramids. By understanding how social structure and age covary, conservationists may be able to implement management practices that promote socio-ecological outcomes and therefore support the viability of wild populations. Vital rates (e.g. survival, reproduction) are integral to understanding population trajectories, and particularly in populations with strong social components to their survival and conservation (such as gorillas [90,91] or elephants [92,93]), skews in the age pyramid could drive nonlinear dynamics that might be important to consider. For example, given that older elephants are crucial founts of knowledge concerning the location of valuable resources [94–96], and of imparting this knowledge to others [40], their loss (e.g. through preferential hunting of the old, which is a common phenomenon both in elephants [92,93] and other animals [97]) could have potential ramifications for the conservation of the population as a whole. This is one possible example that serves to illustrate conceivable benefits of building understanding around this topic for applying knowledge to certain scenarios.

### (c) Fitness and evolution

Naturally, ageing has long been considered tightly linked to survival. A key finding across papers in this Special Issue is the close link between social processes and longevity [8,27,29,32]. The cumulative evidence is that some social changes that occur with age may generalize across populations and species. One such pattern is ageing causing an overall decrease in the amount of social associations an individual holds, with this Special Issue alone including studies showing this pattern in insects [15,30], birds [8,27], ungulates [28] and primates [8,46]. Yet whether these social ageing phenotypes impact upon the fitness of individuals and can, or have, evolved remains an outstanding question of critical importance. Indeed, studies directly linking the pace at which an individual’s social behaviour changes with age to a proxy of fitness remain rare.

Further, in many species, the social ageing of an individual may not just alter their own survival or reproductive success, but also that of their associates. The overall age composition and social connectivity of the population may shape the costs and benefits of individual-level social behaviour. For instance, in this Special Issue, Cook *et al.* [33] find that sociality in female forked fungus beetles (*Bolitotherus cornutus*) is under positive selection primarily when the population is comprised mainly of old individuals. Across many social species, older individuals often can play important roles as their social behaviours change in response to their accumulated knowledge and experience, which can enhance the fitness of others. When those others are relatives, the older individual can benefit via inclusive fitness [98–100]. Teasing apart the links between social ageing and fitness can be challenging due to complex feedback loops between social behaviour, the pace of ageing, and reproductive and survival outcomes [31,32,40], all of which will be further exacerbated when comparing across species [101]. But, understanding how changes in sociality over an individual’s lifetime affect both their own fitness and that of others is an important part of understanding how natural selection acts on social ageing.

To fully evaluate the evolutionary pathways of social ageing, and social ageing traits, we must give due consideration to the ‘selection shadow’. In the context of ageing biology, the selection shadow refers to the decline in the force of natural selection with increasing age [102]. As individuals age, their probability of survival decreases, and they contribute less to future generations, leading to weaker selection pressures against deleterious mutations that manifest later in life [52]. In this context, this weakening of natural selection may allow social senescence effects to persist in older individuals, and in species where social networks span multiple generations this phenomenon may influence the evolution of social ageing traits in even more complex ways. Studies in this Special Issue touch on how the selection shadow may manifest in long-lived social species, suggesting that its effects may be more nuanced than previously thought and related to emerging social processes such as disease spread [8,28], but more work directly in this area is now needed [5].

Long-term, multigenerational studies are crucial for understanding the evolutionary impacts of social ageing. These systems enable us to examine how social inheritance and the extended roles of older individuals influence fitness across generations. As Gordon [15] and Campos *et al*. [30] highlight in this Special Issue, exploring these dynamics offers rich opportunities for future research. Understanding these long-term processes will provide valuable insights into how social ageing and fitness are linked not only within an individual’s lifespan but across generational timescales. This multigenerational perspective is essential for elucidating the complex interplay between ageing, sociality and evolution, and for developing a more comprehensive understanding of biological processes.

## 4. Challenges and considerations

Understanding the drivers of social ageing is a clear current challenge. Indeed, the drivers of social ageing are likely multi-faceted, dynamic and sometimes even synergistic or cumulative. They also span biological levels of explanation [1], from senescence of bodily systems to adaptive responses, to changes within an individual or to their environment and to ontogenetic or evolutionary outcomes [4]. Predictions aimed at allowing researchers to explain social ageing in adults were laid out by Siracusa *et al*. [4], yet limits to our understanding of ageing within and outside the social realm make generating predictions challenging. For example, the patterning of reproductive value with age is complex [32] and more information is needed before clear-cut predictions about how changes to reproductive value with age might explain observed patterns of social ageing [4]. Although the focus of this Special Issue is social ageing, one hope is that by promoting a systems-level approach, we encourage others across various disciplines to take up the challenge of fundamental ageing research more generally.

Even with maximally informed predictions to explain social ageing, inferring one explanation of the drivers over another is often not possible from behavioural data alone. Many predictions for behavioural changes with age are similar across divergent explanations. For example, declines in bodily systems, be they motoric, energetic or cognitive, predict declines in social behaviour with age [4,46]. Getting to grips with these explanations has required, and will continue to require, an approach that draws from diverse perspectives, including ageing biology, health research, neuroscience, anatomy, physiology, molecular biology, evolution, ecology and demography [2,5].

When considering the current challenges surrounding furthering our understanding of age and society, it should be noted that although ageing is itself an ontogenetic process, it is not uniformly expressed across an organism’s lifespan and the pace at which ageing occurs can both modify social processes and be modified by them [76,103]. Distinguishing between cause and effect when it comes to social behaviour and ageing is thus a considerable challenge that will require carefully designed longitudinal studies and experimental manipulations that can determine if ageing is followed by a change in social behaviour, or if social behaviour is followed by a change in an organism’s biological age, in addition to detecting feedback loops that may exist between ageing and sociality. Comparative studies on species or populations faced with divergent environmental conditions will also allow researchers to scratch the surface of causality in greater depth. Comparative data that are findable, accessible, interoperable and reusable (i.e. F.A.I.R [104]) are currently rare but efforts are growing in behavioural ecology and demography to create, promote and use such databases [20,21,38,105]; the possible utility of these approaches, particularly in social network ecology, is only just beginning to be widely appreciated [37].

Fundamental rules in social ageing research will also aid in pushing this field forward and will facilitate future comparative endeavours. One such rule should relate to defining the very topics at hand: sociality and ageing. Sociality is multi-faceted in nature, with different interactions, relationships, structures and organisations being expressed across systems. As such, care should be taken to ensure that comparisons are being made at the same level of sociality and that behaviours are selected based on their functional equivalency [21,106]. Sociality may not necessarily simply reduce or change with age but may shift in multiple dimensions (e.g. when individuals have fewer connections but those connections are stronger). Network analysis can distil multidimensional objects into analysable metrics [107], but researchers should take care not to lose important dimensions that might be indicative of social ageing when they do so or lose sight of the generative processes from which they arise. Meanwhile, although the definition of ageing might seem more straightforward than that of sociality, there are pitfalls and facets to consider here as well. To study ageing, studies typically evaluate changes to an individual’s phenotype across its lifespan, often considering ‘chronological age’, i.e. the amount of time that has passed in an organism’s life. Cross-sectional studies that detect a relationship between a phenotype and ‘age’ must be careful not to make inferences about ‘ageing’, especially when they have not ruled out other viable explanations for their results, such as cohort differences, selective disappearance, or terminal investment. In other words, differences between young and old individuals are not necessarily evidence of changes that occur with ageing. Clarity of purpose regarding these potential pitfalls will help future researchers to detect broad-scale patterns across social ageing studies. In addition to chronological age, individuals can vary in their biological ages. For example, some 40-year-olds may have the cardiovascular profile of someone in their 20s, while others may suffer from early-onset cardiovascular disease. The different ‘hallmarks’ of biological ageing, which include molecular, physiological, and physical domains, have received a lot of attention in human health research [6,52] and will be helpful to consider by those hoping to generate a new ecological and evolutionary understanding of social ageing.

Finally, while there are many advantages of using natural systems for understanding age and society, one remaining and potentially increasing challenge is that these animal populations are subject to constant change and disturbance, in ways that could continuously alter the relationship between ageing and sociality. Specifically, along with the natural loss and addition of individuals into the population, anthropogenically driven changes to the environment and direct effects on the populations may be expected to alter biotic interactions in many—often unpredictable—ways. Nevertheless, while such changes are certainly a challenge, they also point to the importance of quickly gaining an understanding of the fundamental rules underpinning how the relationship between ageing and social behaviour can shape the resilience and functioning of a society.

## 5. Summary and future directions

This Special Issue highlights the role of age in shaping social systems across a diverse range of natural populations. The research herein integrates perspectives from evolutionary biology, behavioural ecology, comparative biology and demography, underscoring the diverse ways in which ageing influences individuals, their social connections and the structure of societies. From mechanisms driving within-individual changes to broader evolutionary and ecological outcomes, these studies shed light on how ageing is intertwined with social behaviour and societal function.

The major takeaways from the Special Issue are the advances in understanding how ageing influences social behaviour and societal structures across species, rather than building understanding of a single group of species alone. The research emphasises the general concept of social ageing [5], illustrating how changes in individuals’ social connections as they age may occur [8,27], and how these changes can impact not only their own fitness [30] but also the dynamics of the populations [8,28,40]. By examining these themes across diverse taxa—from insects to birds and mammals—this Special Issue provides a new understanding of how ageing and sociality interact across species and contexts.

Looking ahead, several promising avenues for advancing our understanding of age and society are highlighted throughout the Special Issue. One particularly captivating direction which can enable interdisciplinary collaborations is the comparison between human and other animal populations [5]. Drawing parallels between social ageing in humans and non-human animals offers a useful framework for interdisciplinary understanding of ageing across species boundaries. As drawn on throughout the Special Issue, animal populations can act as model systems for developing fundamental understanding of the interplay between age and society. Other applied areas would also benefit from such advances; conservation strategies, for instance, could incorporate knowledge about how ageing impacts social structure, fitness and resilience in endangered populations and building an understanding of social ageing in conservation-relevant systems may further our understanding of the ecology of age and society [3]. Moreover, as species face increasing environmental challenges, understanding how social ageing interacts with demographic shifts, such as declining birth rates and skewed age structures, will be important for mitigating potential negative outcomes. Future research should now include a focus on cross-species comparisons and the development of models that account for both ecological and evolutionary processes governing social ageing.

As a final note, it is abundantly clear that long-term studies will remain key tools for deepening our knowledge of social ageing. As emphasized throughout this Special Issue, these studies not only provide insights into the natural progression of ageing but also offer opportunities to explore creative methodological approaches within established settings with known ecologies. More baseline funding and support for longitudinal research will be useful for ensuring that we can continue to unravel the complexities of ageing in natural populations. By connecting insights from various systems and approaches, this Special Issue lays the groundwork for a more integrative understanding of ageing and society. It highlights the need for continued interdisciplinary research that bridges behavioural, ecological and evolutionary perspectives to illuminate how ageing shapes the social fabric of animal societies.

## Data Availability

This article has no additional data.

## Dedication

Dedicated to Allan M. Firth 1935–2024.
